# Combined Motor and Cognitive Rehabilitation: The Impact on Motor Performance in Patients with Mild Cognitive Impairment. Systematic Review and Meta-Analysis

**DOI:** 10.3390/jpm12020276

**Published:** 2022-02-14

**Authors:** Pawel Kiper, Michelle Richard, Françoise Stefanutti, Romain Pierson-Poinsignon, Luisa Cacciante, Cecilia Perin, Miryam Mazzucchelli, Barbara Viganò, Roberto Meroni

**Affiliations:** 1 Physical Medicine and Rehabilitation Unit, Azienda ULSS 3 Serenissima, 30126 Venice, Italy; 2 Department of Physiotherapy, LUNEX International University of Health Exercise and Sports, L-4671 Differdange, Luxembourg; m.richard.kine@gmail.com (M.R.); francoisestefanutti@gmail.com (F.S.); romain_pierson@yahoo.com (R.P.-P.); roberto.meroni@lunex-university.net (R.M.); 3 Laboratory of Rehabilitation Technologies, IRCCS San Camillo Hospital, 30126 Venice, Italy; luisa.cacciante@hsancamillo.it; 4 School of Medicine and Surgery, University of Milano-Bicocca, 20126 Milan, Italy; cecilia.perin@unimib.it (C.P.); m.mazzucchelli@campus.unimib.it (M.M.); 5 GDS Foundation, 20841 Carate Brianza, Italy; barbaravigano1@gmail.com; 6 Luxembourg Health & Sport Sciences Research Institute A.s.b.l., L-4671 Differdange, Luxembourg

**Keywords:** MCI, combined training, motor and cognitive training, motor performance

## Abstract

Mild cognitive impairment (MCI), a neurodegenerative disease leading to Alzheimer’s disease or dementia, is often associated with physical complaints. Combined physical and cognitive training (PCT) has been investigated to see the effects on cognitive function, but its impact on motor functions and activities of daily living has not been explored yet. The combination of physical and cognitive training may be a valuable non-pharmacological intervention that could preserve motor function and quality of life (QoL). We aimed, therefore, to analyze if combined PCT is effective at improving motor performance in patients with an MCI. A systematic electronic literature search and a meta-analysis were conducted. The following criteria were compulsory for inclusion in the study: (1) randomized controlled trial design; (2) combined PCT compared to motor training alone or no intervention; (3) motor outcomes as a study’s end point. Nine articles met the inclusion criteria. Results showed that PCT significantly enhances balance compared to motor training alone (SMD 0.56; 95% CI 0.07 to 1.06; I^2^ = 59%; 160 participants), whereas a significant improvement was found for mobility in the PCT group when compared to no intervention (MD −1.80; 95% CI −2.70 to −0.90; I^2^ = 0%; 81 participants). However, there is no evidence that people with MCI experience an increase in gait speed and QoL at the end of their practice sessions. Further investigation with larger samples and a longer period of monitoring after intervention should be undertaken.

## 1. Introduction

### 1.1. Description of the Condition

The aging rate of the worldwide population is increasing, generating an increase in the risk of developing neurodegenerative diseases and cognitive impairments [1]. Mild cognitive impairment (MCI) can lead to Alzheimer disease (AD) or neurodegenerative diseases [2]. The incidence rates of MCI estimated from a recent meta-analysis per 1000 person-years were 22.5 (5.1–51.4) for 75–79 years, 40.9 (7.7–97.5) for 80–84 years, and 60.1 (6.7–159.0) for 85+ years [3]. MCI is widely regarded as a combined change in memory and cognition that is greater than expected for an individual’s age and education level, but which does not interfere with Activities of Daily Life (ADL) [4]. It is frequently associated with an increased risk of dementia, especially when it is diagnosed with apathy, and it seems to predict which patients with amnestic MCI will progress to AD [5]. Indeed, MCI is classified in two types: amnestic (memory deterioration) and non-amnestic (cognitive function impairment) [6]. The progression of MCI to Alzheimer’s disease and dementia is estimated to be 10–15% per year [7]. Dementia in 2010 touched 35.6 million people worldwide and will rise significantly in the coming years: the figure is estimated to be 65.7 million by 2030 [8]. The associated costs and disease burden have exerted significant pressure on economic and social systems [9]. Thus, research into treating or preventing conditions of age-related neurodegenerative diseases is an urgent public health priority.

In order to establish diagnostic indicators for MCI, Albert et al. developed two sets of criteria. The first one refers to the core clinical criteria that could be used by therapists without access to advanced imaging techniques or cerebrospinal fluid analysis (i.e., evidence of a change in cognition, evidence of an impairment in one or more cognitive domains, the preservation of independence in functional abilities and no evidence of dementia). The second one refers to criteria that incorporate the use of biomarkers based on imaging and cerebrospinal fluid measures [10].

Furthermore, some screening tools could be used for referring individuals to a more thorough examination. Some of these include the Montreal Cognitive Assessment [11] and the Mini-Mental State Examination (MMSE) test, which is the best known and the most often used short screening tool for providing an overall measure of cognitive impairment in clinical settings [12]. However, recent research has showed that both cognitive and functional measures should be combined to assess global functioning in older adults with cognitive impairment, considering cognitive and functional measures as part of one single dimension (i.e., global functioning), which can be measured as a single construct and score [13].

Clinical responses to pharmacological treatments (e.g., donepezil, galantamine, rivastigmine) are insufficient or ineffective on patients with MCI [14]. Thus, there is a need to develop non-pharmacological therapeutic approaches for this disease.

Cognitive stimulation is the most recommended non-pharmacological approach for cognitive symptoms in MCI and for mild-to-moderate dementia [15]. Indeed, a Cochrane review that focuses on interventions within this field concluded that general cognitive stimulation and reality orientation approaches consistently produce improvements in general cognition and, in some cases, in self-reported quality of life (QoL) and well-being, primarily for people with mild-to-moderate dementia [16]. Furthermore, a recent review focusing on the use of the Cognitive Stimulation Therapy (CST) suggests that the use of this method provides evidence of its efficacy for the improvement of general cognitive function, language comprehension/production, and quality of life in patients with mild-to-moderate dementia [17].

Physical training (PT) to prevent dementia and to delay cognitive decline have gained considerable attention in recent years. PT is composed of endurance training and strength, balance, and flexibility exercises. These training sessions are intended to improve strength, flexibility, cognition (e.g., attention, memory, and executive function), aerobic capacity, and balance [18]. 

Aerobic exercise has demonstrated significant improvements in global cognitive scores with a weak but significant effect on memory [19]. Evidence suggests that regular aerobic exercise acts as a promoter of “brain health”, mediating neural homeostasis and, through neuroprotective and neurorestorative mechanisms, counteracts brain ageing [20]. At the behavioral level, aerobic exercise has been found to upregulate affective states [21,22], and to improve cognition throughout different age phases [23,24] and different dimensions, including spatial/associative learning [25,26], attentional processing [27], and executive control [28].

It has also been demonstrated in several studies that some activities, such as PT, being engaged in the stimulation of cognitive activities, the maintenance of active social life into old age, and the control of nutrition, are correlated with maintaining good brain function, particularly in the elderly, and reduce the risk of developing dementia [29].

In the same way, Bamidis et al. showed that dual tasks can produce new neuronal networks or strengthen synaptic activity [30]. 

Moreover, most of the ADL require dual task performance, a combination of motor and cognitive functions which involve high and complex demands [18]. The performance of ADL, due to their complexity, is more difficult to realize for people with neurodegenerative disease [31].

Dual tasking is not only required for ADL, but also for the training of physical and/or cognitive skills [18]. According to Schaefer and Schumacher, PT combined with CT improves physical and mental abilities [32]. 

### 1.2. Description of the Intervention

The studied intervention of this review is the combination of physical and cognitive training (PCT) in people with MCI.

We considered PCT as a planned and constant practice of motor and cognitive exercise with the purpose of sustaining or improving performance. CT included in this review targets multiple cognitive domains, such as attention, memory, orientation, executive function, visual and auditory tasks, and social interaction.

PT included aerobic exercise, exercises to improve muscle strength, physical function, balance control, and flexibility.

Aerobic exercises are mainly conducted on a cycle ergometer. Balance was trained in both static and dynamic modalities by using a balance board or by walking in certain conditions (e.g., over obstacles, by changing direction, walking on different supports). Flexibility exercises were generally performed in combination with relaxation, and physical functions were trained for the upper limb by Simionatto et al. [33] by completing puzzles, painting, drawing, playing dominoes, and more.

Finally, dual task training was also included, as it combined PCT. In the study by Shimada et al. [34], participants played word games while doing stepping exercises.

### 1.3. Why It Is Important to Do This Review

The effects of combined PCT for the improvement of motor functions (mobility, balance, gait speed, gait endurance, upper limb functions), as well as its implications on QoL and ADL in people with MCI, have not been extensively evaluated and summarized in the frame of a comprehensive review.

As many of the previous reviews focused mainly on cognitive improvements, this review focuses on motor improvements following a combined PCT. Thus, this will provide insights for physiotherapists and other clinicians about possible approaches for a comprehensive treatment for MCI patients.

### 1.4. Objectives

#### 1.4.1. Primary Objective

The primary aim of this systematic review was to analyze whether combined PCT has an impact on motor functions in MCI patients, as compared to PT alone or no treatment.

#### 1.4.2. Secondary Objectives

The secondary aim was to assess if combined PCT influences QoL and/or ADL.

## 2. Materials and Methods

The study design was set as a systematic review and meta-analysis and was conducted according to the PRISMA guidelines [35]. The protocol was registered a priori in the PROSPERO database under the following registration number: CRD42020201928.

### 2.1. Criteria for Considering Studies for This Review

#### 2.1.1. Types of Studies

We included studies with the following criteria:

- Randomized controlled trial (RCT) or quasi-RCT, controlled clinical trial, or case–control study;
- Patients with MCI (diagnosed by psychologists or psychiatrists, based on criteria proposed by European Consortium on Alzheimer’s Disease Working Group on MCI or with standard clinical examinations in line with the criteria of ICD-9-CM);
- Studies investigating MCI and motor impairments;
- Interventions must involve combined PCT compared to PT alone, no intervention, or placebo;
- Outcomes include motor functions (mobility, balance, gait speed, gait endurance, upper limb functions).

We excluded studies with the following characteristics:

- Study design set as case report, review, study protocol, or case series;
- CT provided alone or PT provided alone as main intervention;
- All forms of telerehabilitation;
- All outcomes not related to motor function domains.

#### 2.1.2. Types of Participants and Interventions

We included studies with adults affected by MCI. Children and healthy adults, as well as people with other diseases not related to MCI (e.g., schizophrenia, autism spectrum disorders, dementia, depression, Alzheimer’s disease, stroke, Parkinson’s disease) were excluded.

We included studies that reported a comparison between combined PCT as the experimental treatment versus a control intervention consisting of PT alone, no intervention, or a placebo. 

#### 2.1.3. Types of Outcome Measures 

The outcomes included were outcomes related to motor functions, to the perceived QoL, and to the ADL. Additionally assessed were improvements of motor functions, with outcome measures assessing gait speed and endurance, balance, mobility, and upper limb functions. In addition, the improvement of perceived QoL and ADL were evaluated through questionnaires.

### 2.2. Search Methods for Identification of Studies 

#### Electronic Searches

A systematic electronic search of the literature was carried out online through PubMed, Scopus, Embase, Web of Science, and the Cochrane Library. The search strategy was conducted through the creation of different search syntaxes, based on the guidelines of each database, with the following terms: (“Cognitive impairment” OR “Cognitive disorder” OR “Neurocognitive Disorders” OR “mild cognitive impairment”) AND (“motor treatment” OR “physical therapy”) AND (“Cognitive exercise” OR “Cognitive training” OR “Neuropsychological treatment”) AND (“motor function” OR “Activities of Daily Living” OR “upper limb motor function” OR “posture” OR “balance” OR “lower limb motor function” OR “gait speed”).

Only studies published in English were considered. The final database search was run on 1 July 2021 (Appendix A).

### 2.3. Data Collection and Analysis

#### 2.3.1. Selection of Studies

For the abstract screening, six reviewers were divided into three groups (i.e., two reviewers for each group). Records retrieved were divided equally into the three groups. The reviewers independently screened records that were identified, based on title and abstract, using an inclusion/exclusion criteria template. A third reviewer was assigned to each of the three groups to solve any disagreements. At the end of this process, the full text of the articles was obtained, and the same procedure was used for full text screening and for the assessment of the methodological quality of the studies.

#### 2.3.2. Data Extraction and Management with Missing Data

We extracted information related to methods, participants, interventions, outcome measures, and conclusions drawn from authors, as well as the year of publication, study design, number of participants, groups characteristics, and information related to the adherence to interventions.

#### 2.3.3. Assessment of Risk of Bias in Included Studies

The Revised Cochrane risk-of-bias tool for Randomized Trials (RoB2) was used to evaluate the methodological quality of the included studies. 

Five domains were assessed: (a) Selection bias, (b) Performance bias, (c) Detection bias, (d) Attrition bias, and (e) Reporting bias. For each domain, the risk of bias was coded into one of the three following possibilities:

- Low: low risk of bias.
- High: high risk of bias.
- Some concerns: when the reporting was insufficient, and some concerns were raised.

#### 2.3.4. Measures of Treatment Effect 

We used Review Manager 5.3 to conduct the review and for statistical analysis. The treatment effects were evaluated using the Mean Difference for homogeneous outcome measures or the Standardized Mean Difference (SMD) for the outcomes evaluated with different scales. The Confidence Interval (CI) for continuous outcomes was identified at 95%.

#### 2.3.5. Assessment of Heterogeneity 

Statistical heterogeneity was assessed with the I^2^ statistic, establishing the cut-off value at 50%. We conducted the meta-analyses based on the random effects model or fixed effects model with 95% CI. 

## 3. Results

### 3.1. Description of the Studies

#### 3.1.1. Results of the Search 

**Figure 1:**
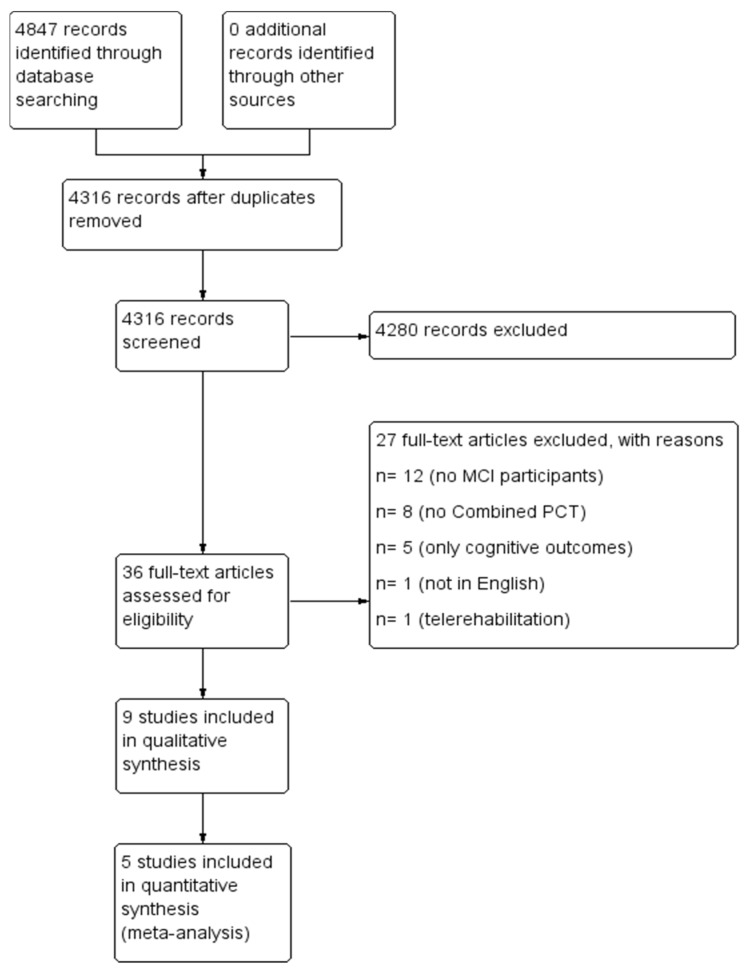
PRISMA Flow Diagram.

A total of 4847 articles via electronic searching were retrieved. After removing the duplicated records, 4316 records remained for abstract screening. After abstract screening, 36 articles were included for full text reading. Finally, nine papers were included for qualitative evaluation and six studies were quantitatively analyzed (Figure 1). 

#### 3.1.2. Excluded Studies

After full-text screening, 27 articles were excluded. Twelve of them were excluded due to the inclusion of non-MCI participants, for eight studies the intervention was not defined as combined PCT, and five of them were not included because only cognitive outcomes were assessed. One study was excluded due to the use of the Spanish language, and another one was excluded due to different kind of intervention provided (i.e., telerehabilitation program) (Figure 1).

#### 3.1.3. Included Studies

The characteristics of the included studies and related interventions are presented in Appendix B and Appendix C.

Cintoli et al. [36] investigated the effectiveness of a combined PCT program in patients with MCI. Neuropsychiatric symptoms via the Neuropsychiatric Inventory and QoL via the QoL-Alzheimer’s disease (QoL-AD) measures were assessed at baseline and at seven months. The results demonstrated significant ameliorations for QoL-AD scores, which increased (*p* = 0.0013) for the training group. The authors concluded that this strategy could improve QoL and possibly prevent early cognitive decline and its consequences.

Combourieu Donnezan et al. [37] aimed to study the effects of aerobic plus CT together, as well as single training for executive functions and cardiovascular and functional capacities. Participants were randomized into four groups, in which neuropsychological tests, the Rockport test for cardiovascular fitness, and walking tests were provided. Outcomes were measured at baseline and six months after the training programs. Results demonstrated significant improvements for executive and walking capacities, especially for the PCT program (TUG, *p* < 0.001; Rockport test, *p* < 0.05), where eight outcomes demonstrated significantly greater scores, with these results lasting over time also. It was concluded that simultaneous PCT is more convenient when combined rather than when isolated, as MCI patients will benefit, especially for motor capacities.

Hagovská (A) [38] evaluated the efficacy of the CogniPlus program combined with balance training for postural and functional status in seniors with MCI. The authors assessed at baseline and ten weeks later cognitive status with the MMSE and with the Addenbrooke’s cognitive examination. In addition, they assessed postural control and functional activities using balance tests and questionnaires. The intervention group showed significant improvements for cognitive and balance capacities (*p* < 0.05–0.0001), but functional activity did not show significant changes. Combined PCT allowed greater improvements in both cognitive and balance activities.

In another paper, Hagovská et al. (B) [39] implemented the same intervention as the one mentioned above, but this time assessing gait, fear of falling (FoF), and QoL in seniors with MCI. MMSE was used for cognitive evaluation, FoF was evaluated through the Falls Efficacy Scale I. The Tinetti test was used for balance assessment, Timed Up and Go (TUG) for gait, and Spitzer for QoL. All these outcomes were measured at baseline and ten weeks after the baseline. Significant differences were observed for MMSE, TUG with dual tasking, the Tinetti test, and the QoL questionnaires (*p* < 0.03–0.0001), but not for the Falls Efficacy Scale I. The combined intervention appears to be more effective than single training.

Hagovska et al. [40] once again studied the same intervention of combining the CogniPlus program with physical–balance training and measured its effectiveness on cognition and ADL. The authors added several cognitive outcomes on top of the MMSE and used the Bristol-ADL Scale to assess ADL. Significant improvements were observed for ADL (*p* ≤ 0.0001, effect size = 0.176). Combined training seemed to be more effective for cognitive and ADL outcomes, rather than training alone.

Lipardo et al. [41] evaluated the impact of combined PCT on fall rate and risks of falling in older adults with MCI. Three groups were settled, with two of them including either PT or CT alone, and one including combined PCT. Assessments were conducted at baseline, twelve, and 36 weeks after baseline by using fall incidence, the Physiological Profile Assessment Short-Form for risk of falls, the TUG, the 10 m Walking Test, and the 30 s Chair Stand Test. Significant differences were only observed on the TUG in the combined PCT group (*p* = 0.001) and cognitive group (*p* = 0.012). Combined PCT did not change fall incidence and risks of falling, but enhanced dynamic balance.

Middleton et al. [42] analyzed a mental activity and exercise trial which combined mental activity and exercise programs and assessed its effects on physical function and QoL in people with cognitive complaints. Four groups were randomized in combined training programs, either mental or exercise alone, and in a control group. The assessments took place at baseline and twelve weeks afterwards by using the Senior Fitness Test for physical function and the Short-Form-12 health survey for health-related QoL. Significant differences were noted on physical function for many measures (e.g., chair stands, *p* = 0.001; sit and reach, *p*-time = 0.01) and in health-related QoL (*p*-time = 0.04), but not for the TUG. Physical and mental activities together may be beneficial for physical function and health-related QoL, as modified physical activity is linked with physical function and health-related QoL changes.

Shimada et al. [34] studied how cognitive and mobility functions can be affected by a combined PCT program, and compared it with a health education program in an MCI population. MMSE, Wechsler Memory Scale-Revised-Logical Memory II, and the Rey Auditory Verbal Learning Test were performed for cognitive assessment. Furthermore, MRI and mobility evaluations were conducted at baseline and after 40 weeks. All cognitive outcomes demonstrated significant improvements (MMSE, *p* = 0.012; WMSRLM, *p* = 0.004), except for the RAVLT. Additionally, better mobility scores were observed. Amnestic MCI patients seemed to benefit from combined PCT in terms of cognitive and physical activity.

Finally, Simionatto et al. [33] studied whether functionality and cognition were influenced by manual motor function and CT in the elderly. Combined PCT training was delivered, and the effectiveness was assessed at baseline and after intervention by using the Katz Scale, Barthel index, MMSE, Clock-Drawing Test, and Verbal Fluency test. The MMSE, Katz Scale, and Verbal Fluency Test showed significantly greater scores after intervention in the intervention group (*p* < 0.05). This way of using sensory motor plus CT might be helpful for institutionalized elderly in terms of functionality and maintaining adequate QoL.

### 3.2. Risk of Bias in Included Studies 

**Figure 2:**
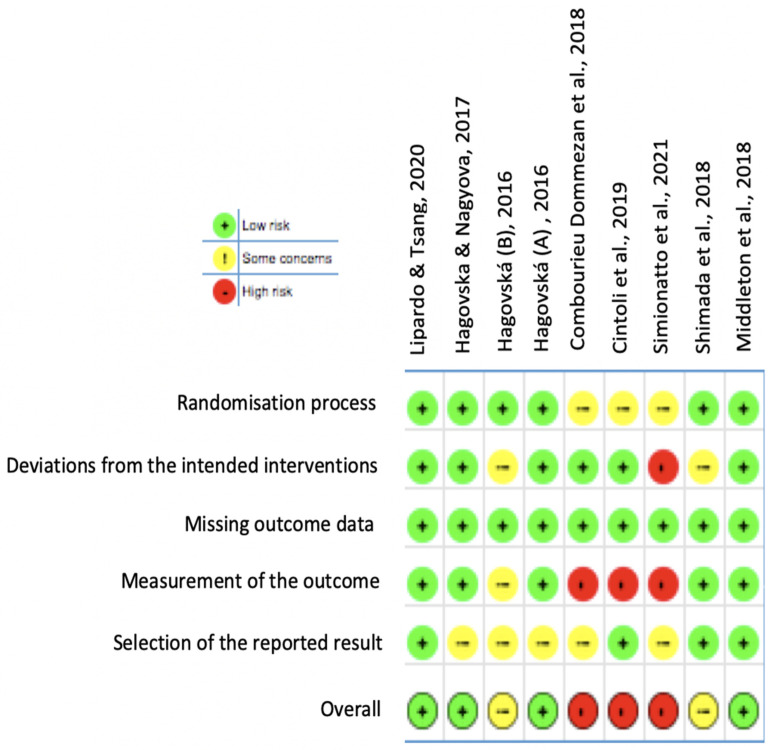
Risk of bias summary: review authors’ judgments about each risk of bias item for each included study.

The risk of bias of the included studies is displayed in Figure 2 and presented in more detail in Appendix D. The evaluation found the following results:

➢ Randomization process and allocation concealment (selection bias): Almost all studies were judged to have a low risk of bias. Studies by Combourieu Donnezan et al., Cintoli et al. and Simionatto et al. were found to have some concerns, as concealment is not mentioned and randomization is not detailed.
➢ Deviation from intended intervention (performance bias): The studies by Hagovská (B) and Shimada et al. raised some concerns about the presence of bias, and Simionatto et al.’s study was classified as high risk due to absence of information about participants’ withdrawing, and no intention-to-treat analysis was performed. The six remaining studies showed a low risk of bias.
➢ Missing outcome data (detection bias): A low risk of bias was found in all the included studies.
➢ Measurement of the outcome (attrition bias): The study by Hagovská (B) was judged to raise some concerns. As Cintoli et al. (2019) did not provide information for outcome assessors and assessors’ knowledge of intervention received and Simionatto et al. (2021) did not detail the timeline of assessment and assessors were aware of intervention received, they were judged to have a high risk of bias. Furthermore, the study by Combourieu Donnezan et al. raised high risk of bias as measurement of the outcome have differed between intervention groups. The other five studies showed a low risk of bias.
➢ Selection of the reporting result (reporting bias): The studies by Hagovska and Nagyova, Hagovská (B), Hagovská (A), Combourieu Donnezan et al. and Simionatto et al. were evaluated to have some concerns, whereas the four remaining studies were assessed to have a low risk of bias.

### 3.3. Effects of Intervention—Combined PCT Compared to Motor Treatment Alone

Five studies were included for meta-analysis on the effect of combined PCT related to the mobility, balance, gait speed and ADL outcomes in patients with MCI [37,38,39,40,41], with a total of 401 participants. 

#### 3.3.1. Mobility

**Figure 3:**
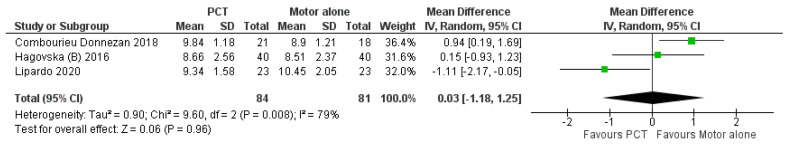
Forest plot of comparison: 1 Motor + cognitive training PCT versus control group Motor alone; Outcome: 1.1 Mobility post-treatment.

Three studies examined the effect of combined PCT on mobility [37,39,41] by using TUG. Analyses were performed with the Mean Difference (MD) and a random effects model. No significant difference was found between the control and intervention group (MD 0.03; 95% CI −1.18 to 1.25; I^2^ = 79%; 165 participants; Figure 3). 

#### 3.3.2. Balance

**Figure 4:**
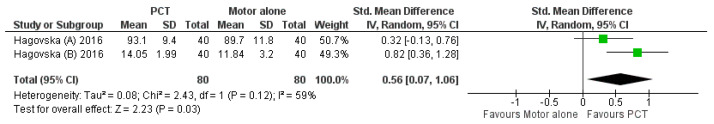
Forest plot of comparison: 1 PCT versus Motor alone; Outcome: 1.2 Balance post-treatment.

Two studies examined the effect of PCT on balance [38,39]. The analyses were performed with the Standardized Mean Difference (SMD) and a random effects model. The results showed that balance improved significantly in the combined PCT group compared to the PT group (SMD 0.56; 95% CI 0.07 to 1.06; I^2^ = 59%; 160 participants; Figure 4). However, the analysis showed high heterogeneity.

#### 3.3.3. Gait Speed

**Figure 5:**
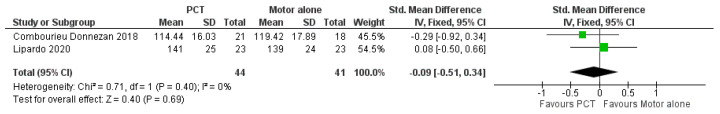
Forest plot of comparison: 1 PCT versus Motor alone; Outcome: 1.3 Gait speed post-treatment.

To assess gait speed, two studies were included [37,41]. Analyses were performed with the SMD and a random effects model, showing no significant effect of combined PCT on gait speed (SMD −0.09; 95% CI −0.51 to 0.34; I^2^ = 0%; 85 participants; Figure 5).

#### 3.3.4. ADL

**Figure 6:**
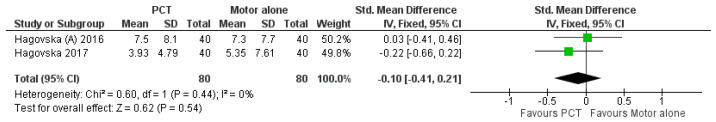
Forest plot of comparison: 1 PCT versus Motor alone; Outcome: 1.4 ADL post-treatment.

When assessing ADL with the SMD and a random effects model, two studies [38,40] showed a similar effect of combined PCT when compared to the control group for ADL (SMD −0.10; 95% CI −0.41 to 0.21; I^2^ = 0%; 186 participants; Figure 6). 

### 3.4. Effects of Intervention—Combined PCT Compared to No Intervention

Data from two studies [37,41] were obtained and analyzed for the comparison between combined PCT and no intervention, and they were assessed for the outcomes on mobility and gait speed. The overall number of participants included in the analyses was 81.

#### 3.4.1. Mobility

**Figure 7:**
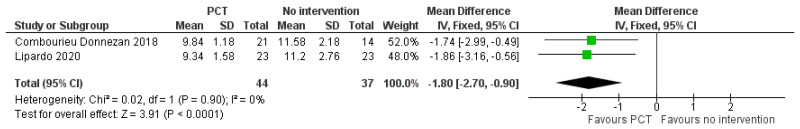
Forest plot of comparison: 2 PCT versus no intervention; Outcome: 2.1 Mobility post-treatment.

The analyses were performed with the Mean Difference (MD) and a random effects model by using TUG. A significant difference was found between the control and intervention group in favor of combined PCT (MD −1.80; 95% CI −2.70 to −0.90; I^2^ = 0%; 81 participants; Figure 7).

#### 3.4.2. Gait Speed

**Figure 8:**
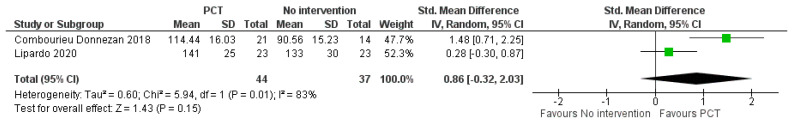
Forest plot of comparison: 2 PCT versus no intervention; Outcome: 2.2 Gait speed post-treatment.

Analyses were performed by using the Standardized Mean Difference (SMD) with a random effects model. No significant difference was found between the control and intervention group (SMD 0.86; 95% CI −0.32 to 2.03; I^2^ = 83%; 81 participants; Figure 8).

Four studies were excluded from the analyses due to insufficient data [33,34,36,42].

## 4. Discussion

### Summary of Main Results and Applicability of Evidence

The present study was designed to determine the effect of combined PCT on motor function, gait, balance, ADL, and health-related QoL. Results from the review showed no evidence of effectiveness and a low effect on motor function in people with MCI immediately after intervention. Nevertheless, combined PCT was associated with better results for balance when compared to motor treatment alone. Conversely, the analyses of combined PCT versus no treatment showed no effect for gait speed, but a significant improvement was observed for mobility in favor of the combined PCT group.

Furthermore, the results need to be considered carefully because of some limitations. Three out of the six analyses showed moderate-to-high values for heterogeneity, highlighting the presence of important inconsistency. The *Cochrane Handbook for Systematic Reviews of Interventions* suggests the consideration of several possible sources of heterogeneity [43]. Indeed, the likelihood of drawing correct inferences from a meta-analysis decreases with increasing heterogeneity [44]; thus, the investigation of and plausible explanations for the presence of heterogeneity are required. The results on mobility and balance (i.e., combined PCT vs. motor treatment alone) as well as gait speed (i.e., combined PCT vs. no intervention) are probably affected by clinical heterogeneity, likely due to differences in types and doses of intervention. In fact, combined PCT reflects a wide variety of intervention programs, and the probability of being faced with very different kinds of training is high. Despite knowing the definition of PT and CT, the studies investigating combined PCT did not use exactly the same intervention across the different articles included in this systematic review. This highlights the fact that rehabilitation programs for patients with MCI need to be personalized and developed in accordance with patients’ needs. Thus, an important issue that arises here is that, on the one hand, there is a need to provide treatment tailored to the individual patient and, on the other hand, there is a need to offer standardized treatments that can be compared in terms of effectiveness. Another limitation that needs to be underlined is that some of our results are based on few studies, resulting in a reduction of the amount of data available for the analyses. This observation highlights the need to encourage researchers to focus on this topic, which is clinically relevant for the aging population. Furthermore, for further research, it would be necessary to have large-scale studies in order to see the impact of the interventions on larger samples, and to have conclusive results.

Undoubtedly, the theoretical premises for a systematic use of combined motor and cognitive training in patients with MCI appear to be of clinical interest. As pointed out in the introduction, both cognitive and motor skills interact within ADL. Therefore, we can observe actions performed in a dual-task manner many times. It is worth noting that when cognitive resources begin to be deficient, it is also likely that daily activities begin to be less efficient. However, in the examined studies in this systematic review, little attention has been paid to this aspect, whereas it was possible to obtain some suggestions related to motor skills. In particular, balance was more influenced by the combined treatment. This result can be due to fact that balance requires greater attention and concentration than, for example, walking speed [45]. Analyses also showed that walking speed compared both to motor treatment alone and no intervention was not inferior to combined PCT, and was thus potentially not affected by this kind of treatment. Interestingly even no intervention was not found to be inferior to combined PCT. However, greater attention must be paid at the interpretation of this result: the high level of heterogeneity, the low number of studies, and the wide confidence intervals leave some doubts for definite conclusions to be drawn from this analysis. Therefore, what is shown in the meta-analysis must be carefully interpreted. 

Having analyzed several tests used to measure cognitive performance in the included studies, it can be seen how tests aimed at assessing attention and concentration (i.e., the Stroop test, the TMTest, the attention and concentration subitems of the MMSE) showed significantly improved scores after training. This provides novel and encouraging information that subjects with MCI can improve their balance through combined cognitive and motor exercises.

If ADL are extremely important for maintaining independence, the perception of quality of life (health-related QoL) is another element affected by good motor and cognitive performance. The difficulty of analyzing health-related QoL arises from the fact that it is a very subjective outcome and requires a global evaluation tool of functioning, such as the International Classification of Functioning (ICF), which is a highly versatile and adaptable assessment tool for every need [46]. Reflecting on the already mentioned issue of personalized versus standardized treatments, we can think about developing rehabilitation protocols for patients with MCI based on ICF qualifiers, which are useful for designing patient-centered care [46], especially when patients have more heterogeneous needs and require more personalized programs. Thus, it is desirable that future RCTs make use of personalized protocols based on universally applicable ICF-guided assessment tools and are directed to the subjects affected by MCI [47].

## 5. Conclusions

(a) *Implications for practice*

Given the fact that we see an increasing number of people with MCI, it is important to explore the different training systems which can potentially improve motor function, such as combined PCT. People with MCI experience not only cognitive difficulties, but also impairments in performing dual tasks that require motor function and cognitive abilities. Large-scale trials should be conducted to investigate the effectiveness of such kinds of training, especially in people with MCI. Interestingly, this review found a high level of adherence to combined PCT for people with MCI, suggesting that this kind of treatment is well accepted by MCI patients and could be implemented in a larger scale. Indeed, given these findings, it could be interesting to develop trials that include an assessment of the satisfaction levels of MCI patients who underwent combined PCT, so as to be able to find personalized interventions and to advise on the use of an effective treatment that is well accepted by patients and with high levels of patient satisfaction.

(b) *Implications for research*

Pharmacological treatments are limited or inefficient at improving motor functions in people with MCI. Studies investigating the impact of combined PCT on cognitive function demonstrate the positive effect of the intervention. However, the findings of these studies on cognitive function have been supported, but not on motor ones [18]. Indeed, our review highlighted the lack of research in this area, and that further research is needed to establish whether combined PCT in people with MCI have favorable results.

Our in-depth review brought up the issue of high heterogeneity in both treatment programs and outcome measurements. However, an open question arises between the development of personalized, patient-centered interventions, which should be the aim of rehabilitation interventions, and the need to develop more standardized protocols, considering patient-centered protocols for combined PCT, in order to try to draw firm conclusions on an emerging training regime for a growing population, such as patients with MCI.

It would be interesting to investigate the impact of combined PCT after a long-term period after intervention (e.g., one year), as well as the effects and potential benefits of PCT on these, given the importance of muscle function in the elderly in everyday life.

Finally, our review suggests some positive results of combined PCT, but there is an urgent need for further large-scale studies of PCT for people with MCI.

### Limitations

A first limitation of this review is the low number of articles included for the meta-analysis: only five articles were included. This can impact the analysis and conclusion of the effect or lack thereof of the intervention on MCI participants. Another limitation can be related to the low quality of reporting in some studies, which made it impossible to include some studies in the analyses. Finally, we included three articles by the same authors, and it was not possible to detect whether the population analyzed in those articles was the same. We could neither confirm nor exclude those articles a priori, so they have all been reported.

## Data Availability

All data are contained within the article.

## Appendix A

PICO research strategy for combined motor and cognitive training on people with MCI.

## Appendix B

Characteristics of included studies.

## Appendix C

Participant’s intervention.

## Appendix D

Assessment of risk of bias (ROB2 tool).
